# Low-Noise Mid-Infrared Photodetection in BP/h-BN/Graphene van der Waals Heterojunctions

**DOI:** 10.3390/ma12162532

**Published:** 2019-08-09

**Authors:** Qin Lu, Li Yu, Yan Liu, Jincheng Zhang, Genquan Han, Yue Hao

**Affiliations:** 1 Key Laboratory for Wide Band Gap Semiconductor Materials and Devices of Education, School of Microelectronics, Xidian University, Xi’an 710071, China; 2 School of Electronic Science and Engineering, Nanjing University, 163 Xianlin Ave, Nanjing 210023, China

**Keywords:** low noise, mid-infrared, tunneling, heterojunction

## Abstract

We present a low-noise photodetector based on van der Waals stacked black phosphorus (BP)/boron nitride (h-BN)/graphene tunneling junctions. h-BN acts as a tunneling barrier that significantly blocks dark current fluctuations induced by shallow trap centers in BP. The device provides a high photodetection performance at mid-infrared (mid-IR) wavelengths. While it was found that the photoresponsivity is similar to that in a BP photo-transistor, the noise equivalent power and thus the specific detectivity are nearly two orders of magnitude better. These exemplify an attractive platform for practical applications of long wavelength photodetection, as well as provide a new strategy for controlling flicker noise.

## 1. Introduction

Mid-infrared (mid-IR) light (3 to 15 μm) covers two infrared atmosphere windows and contains the fingerprints of most common molecular vibrations [1,2]. Photodetection in this spectral region is of great technical importance for applications ranging from material analysis to Lidar [3], free-space telecommunications [4,5]. Two-dimensional materials are emerging as promising mid-IR sensitive materials due to their broad bandgap coverage and flexible integrability [6,7,8]. These advantages provide new opportunities for material/circuit integration such that cryogenic operation of state-of-the-art mid-IR photodetectors is unnecessary [9,10]. Recently, black phosphorus (BP) was rediscovered as a promising material for mid-IR photonics. Long-wavelength light strongly interacts with BP due to its moderate bandgap [11,12,13]. Tremendous efforts have focused on exploring the application of BP for photodetection. The anisotropy and mechanisms of BP’s photo response are well-understood. A BP p-n junction can also be engineered to improve the responsivity [14,15]. Dark noise is another key factor that determines the performance of photodetectors. However, most previous studies have focused on the photo-transistor architecture [16,17,18,19,20], where high 1/f noise reduces sensitivity in terms of high-noise equivalent power and low detectivity. 

To circumvent this problem, we present a new strategy for compressing flicker noise using BP/boron nitride (h-BN)/graphene tunneling junctions. We propose using h-BN as a tunneling barrier to significantly block dark current fluctuations induced by shallow trap centers. To validate this new strategy, we fabricated van der Waals stacked BP/h-BN/graphene tunneling junctions. The flexibility of 2D materials allows them to be used in heterojunctions without the limitations of lattice mismatch or the thermal expansion coefficient. We measured the temperature-dependent scanning photo-current, charge transport, and low-frequency noise spectrum. While the photoresponsivity of the heterojunction at mid-IR wavelengths (up to 4 μm) is similar to that from a BP photo-transistor, the noise equivalent power and thus the specific detectivity are a factor of 100 larger. The results show that the tunneling heterojunction provides an improved sensitivity compared to that of a BP photo-transistor.

## 2. Experiment

### 2.1. Materials and Methods

**Figure 1:**
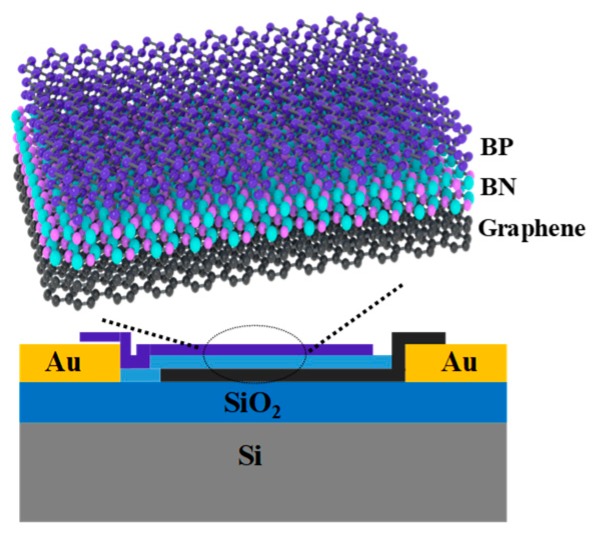
Schematic of the black phosphorus (BP)/boron nitride (h-BN)/graphene van der Waals heterojunction. The few-layer BP flake was transferred onto the few-layer graphene flake and the two were separated with boron nitride, forming a vertically-stacked heterojunction.

Figure 1 shows a schematic diagram of the BP/h-BN/graphene van der Waals heterojunction. A source/drain pattern was defined by a conventional photolithographic process. Ti/Au (5 nm/35 nm) was deposited on a 100 nm SiO_2_/Si substrate using an electron beam evaporator. The materials we used were obtained using a standard mechanical exfoliation process [21]. BP/h-BN/graphene heterostructures were obtained using a dry-transfer technique described in the literature [22]. In the first process, the graphene and h-BN flake were first exfoliated onto a silicon wafer covered with a 300 nm thick thermal SiO_2_ film. With the aid of an optical microscope, polyvinyl alcohol (PVA) was used to pick up these two materials sequentially and transfer them to the defined source electrode to format h-BN/graphene using a deterministic transfer technique. This process was performed on an 80 °C hot plate. The top BP flake on the polydimethylsiloxane film was then dry-transferred onto the h-BN flake and the defined drain electrodes with slight pressure to form the BP/h-BN/graphene vertical heterojunction. The electrical and optoelectrical measurement results of the device were gathered by a semiconductor characterization system. Standard Atomic force microscopy (AFM) data were obtained in tapping mode using a Cypher S AFM. Raman spectra were gathered using a LABRAM-HR Raman spectrometer with an excitation wavelength of 514 nm.

### 2.2. Characterizations

**Figure 2:**
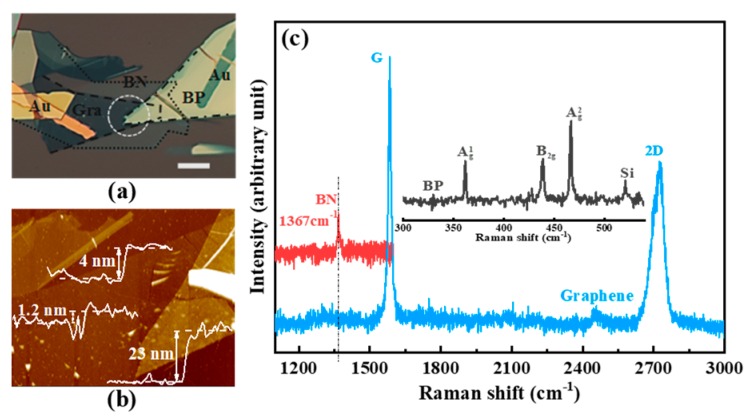
(**a**) An optical microscope image of the black phosphorus (BP)/boron nitride (h-BN)/graphene heterojunction, where the lines indicate different materials: graphene (dashed), h-BN (point), and BP (dot-line). The scale bar is 10 µm. (**b**) Atomic force microscopy (AFM) images of the heterojunction device. The thickness of BP, h-BN, and graphene is ~23, ~4, and ~1.2 nm, respectively. (**c**) Raman spectra of exfoliated graphene (blue), h-BN (red), and BP (black) film in the device on the SiO_2_ substrate.

Figure 2a shows an optical microscope image of a typical BP/h-BN/graphene device, where the dashed lines, points, and dotted line outline the graphene flake, h-BN barrier, and top BP, respectively. The overlapped stacking region is also shown using white lines. Figure 2b shows the corresponding flake heights obtained with AFM. The thickness of the h-BN flake is ~4 nm. We generally selected 3–6 nm sized h-BN, which is appropriate for use as a tunneling barrier. A sample that is too thick (thin) may lead to very low tunneling (very large leakage) under moderate bias. Figure 2c shows three Raman peaks in the BP flake from the inset at ~361, 438, and 466 cm^−1^ corresponding to the A^1^_g_, B_2g_, and A^2^_g_ modes, respectively. These peaks are consistent with previous observations from few-layer BP flakes [23]. Moreover, the low ratio of the Raman D peak indicates that the graphene has a low defect density. The ratio of the Raman 2D to G peak intensities is less than 1 for pristine graphene on the SiO_2_ substrate, indicating that the graphene is only a few layers thick. The additional peak at 1367 cm^−1^ corresponds to the interlayer h-BN film.

## 3. Results and Discussion

### 3.1. Operational Principle

**Figure 3:**
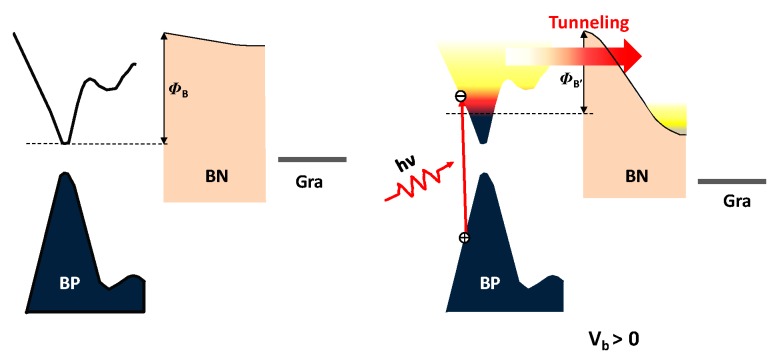
Proposed diagram for the photogenerated carriers in the junction and modulation of the photocurrent at zero bias and positive bias. Φ_B_ and Φ_B’_ are the barrier from boron nitride (h-BN).

The schematic band structure of the BP/h-BN/graphene structure is shown in Figure 3. The insulating h-BN forms a high-energy barrier, preventing the counter graphene electrode from collecting photo-generated carriers in BP. Applying an interlayer bias V_b_ (V_b_ is applied to the top BP with graphene grounded) tilts the band in h-BN, forming a triangular barrier [24]. Increasing V_b_ narrows the equivalent barrier width, allowing photo-excited carriers to tunnel from BP to graphene. As a result, tunneling photocurrent can be generated in the heterostructure device with proper bias and illumination conditions. The photo-carrier transport mechanism is similar to what occurs in a graphene/h-BN/graphene tunneling device; however, optical excitation in BP provides carrier injection here [25].

### 3.2. Electronic Performance of Dark Current

**Figure 4:**
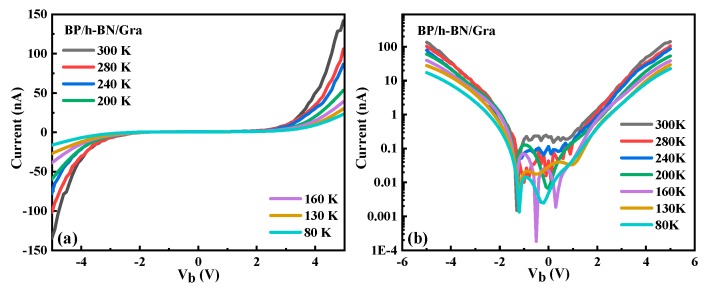
(**a**) Current as a function of V_b_ at different temperatures. (**b**) Logarithmic current-bias plot.

We first characterized the dark I-V performance of the BP/h-BN/graphene structure, as shown in Figure 4. Figure 4b shows a logarithmic plot of the I-V curve shown in Figure 4a. In the case of h-BN, the barrier heights are 3.5 eV for electron transport and 1.3 eV for hole transport [26]. We therefore treat the interlayer transport in an h-BN-based system as dominated by a single carrier type (holes). Considering that mechanically stripped graphene has a Fermi level between 0 and −0.2 eV, the band offset between the conduction band of BN and graphene will be between 1 and 2 eV. Therefore, when the external voltage causes the conduction band at two sides of the heterojunction to tilt by about 1 eV, the tunneling current will increase significantly, which is consistent with the conclusions obtained in our paper. The I-V data indicates a typical Fowler–Nordheim tunneling junction, i.e., the current rapidly increases as V_b_ increases due to the narrowed effective barrier width [27]. A slight difference between the forward and reverse currents is due to the asymmetric device structure. A detailed analysis shows that Fowler–Nordheim tunneling dominates in our device, where a strong electric field allows electrons to tunnel through the barrier. In this regard, the temperature-dependent current is determined by the carrier concentration. The concentration of free charge carriers in the conduction band increases as the temperature increases, which increases the tunneling current [28]. The tunneling current reaches 0.14 μA at 300 K when V_b_ = 5 V, which is a factor 6.36 larger than the current at 80 K (22 nA). We also fabricated a BP photo-transistor as a control sample (see below for details). The current in the BP phototransistor as a function of source-drain bias at different temperatures is summarized in Figures S1 and S2 (Supplementary Information). The equations should be inserted in an editable format from the equation editor.

### 3.3. Photocurrent and Photoresponsivity

**Figure 5:**
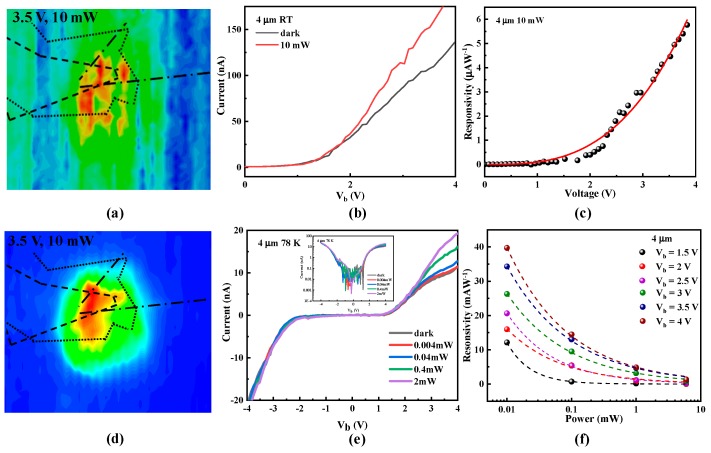
(**a**) Photocurrent mapping of the black phosphorus (BP)/boron nitride (h-BN)/graphene heterojunction device at room temperature with a 10 mW average illumination power and 3.5 V voltage. (**b**,**c**) are the current–voltage characteristic and photoresponsivity of the heterojunction device at room temperature. (**c**) Photocurrent mapping of the BP/h-BN/graphene heterojunction device at 78 K with a 10 mW average illumination power and 3.5 V voltage. (**e**,**f**) are the photocurrent and photoresponsivity in the BP/h-BN/graphene heterojunction device. Source-drain current as a function of source-drain voltage with a 4 μm excitation wavelength with different incident powers detected at 78 K. The inset is a Logarithmic current-bias plot. The data in (**f**) is fitted to a power law I~P_γ_. The dashed lines show fitting results corresponding to each set of data.

We will now discuss the sensitivity of the heterojunction. The photo response and dark noise were measured in order to determine the specific detectivity. Figure 5a,d show maps of the photocurrent from the heterojunction device at room temperature and 78 K with V_b_ = 3.5 V while irradiated with 4 μm light from a 10 mW laser. The top BP layer, h-BN tunneling barrier, and bottom graphene electrode are outlined with a dotted line, point, and dashed lines, respectively. Clearly, the photo-current is generated in the overlap region. Figure 5b,c show the current–voltage characteristic and responsivity for the device measured with a 10 mW incident power under room temperature. Then, we gathered bias and power-dependent photocurrent measurements to further examine the photo-response mechanism and performance.

The dependence of the photocurrent on the incident optical power at 4 μm was measured at 78 K by parking the laser at the centre of the overlap region while sweeping V_b_ at a zero gate voltage. The results are shown in Figure 5e, and the inset shows the curves in Figure 5e on a logarithmic scale. There is no photocurrent at a low bias, but the device produces a high photocurrent as the bias increases. Photocurrent generation can be understood by the aforementioned tunnelling mechanism within the energy diagram shown in Figure 3. The high energy barrier provided by h-BN blocks the transport of photo-excited carriers. A bias between BP and graphene tilts the energy band in h-BN, resulting in a triangular barrier. Increasing the bias narrows the effective width of the energy barrier. Photo-excited charge carriers can then tunnel form BP to graphene, generating a net photocurrent. This working mechanism provides a rational trade-off between dark noise and responsivity. Figure 5f shows the photo-response at 78 K as a function of optical power at 4 μm. The responsivity increases from 1.33 to 39.71 μAW^−1^ at a 4 V applied voltage when the incident power changes from 10 mW to 0.01 mW (4 μm). This negative power dependence of responsivity is similar to that of a BP phototransistor. Besides, a random distribution of defects at the interfaces and vacancies, dislocations, or grain boundaries in BP can result in the formation of shallow trap states [29,30,31,32]. Under illumination, holes (electrons) from generated electron-hole pairs are subsequently trapped, increasing the effective density of carriers in BP. The increased electron density provides a higher tunnelling current driven by the external electric field. However, at an intense illumination, the saturation of trapped shallow traps reduces the overall responsivity. Therefore, although the transport mechanisms differ between our device and that of a BP phototransistor, the photo response is quite similar. Indeed, the photoresponsivity of our device is close to that observed from the BP phototransistor control sample. For example, the calculated mid-IR response at 4 μm reaches 40 μAW^−1^, which is approximately equal to the value of a BP phototransistor control sample (see Figure S3 in the Supporting Information for photocurrent mapping and photoresponsivity measurements from the BP photo-transistor).

### 3.4. Noise Mechanism and Detector Sensitivity

**Figure 6:**
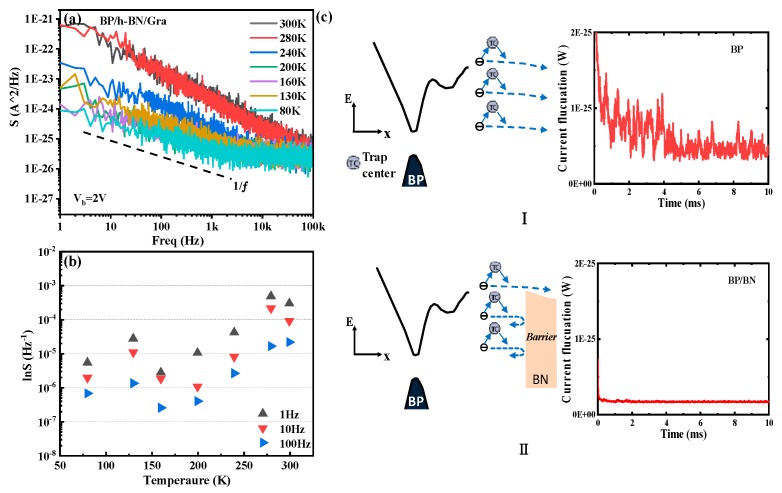
(**a**) Noise spectral density S(I^2^) for the black phosphorus (BP)/boron nitride (h-BN)/graphene device at various temperatures. The source-drain bias was 2 V. Note that the 1/f noise decreases as the temperature decreases. S(I^2^) is two orders-of-magnitude smaller at a relatively low temperature (80 K) than that at room temperature. (**b**) Noise spectral density as a function of the temperature at 2 V bias. (**c**) Energy band diagrams near the BP (I part) and BP/BN (II part) contact representing the current fluctuation probability. The left panel in each part shows carrier transport and the right panel in each part shows current fluctuations in the time domain at the same current values and temperature.

Determining the detector sensitivity requires determining the noise and responsivity. We will see that the tunneling barrier considerably compresses dark noise, resulting in giant room-temperature detectivity, even with a low responsivity. Figure 6a shows the dark current spectral density (SI^2^) at different temperatures. To further validate the appropriate method for reducing noise used here, the dark current spectral density from the BP phototransistor control sample at different temperatures is summarized in Figure S2 (Supplementary Information). Compared with a BP photo-transistor, the sensitivity of the BP/h-BN/graphene heterojunction-based tunneling junction photodetector is ~100 fold larger. Figure 6b shows the noise spectral density as a function of temperature at a low frequency. The noise sensitivity increases as the number of traps decreases at a lower temperature, which indicates that traps are the main noise source. The higher sensitivity can be understood by examining the noise mechanism. It is well-known that the most common description of 1/f noise stems from a superposition of individual generation–recombination noise sources, with the lifetime distributed over a wide timescale, which is usually found at f < 100 kHz in electronics [33,34,35]. Trap centers near the channel-insulator interface are an important type of generation-recombination noise source. As a result, the change in the charge carrier density due to charge trapping/release at trap centers leads to significant current fluctuations. The process is shown in part I in Figure 6c. It is clear that carriers that are captured and emitted back to the channel due to traps at the BP interface significantly contribute to dark noise. We sampled the noise data in the time domain at 80 K to directly characterize drain current fluctuations. On the other hand, the current fluctuation can be effectively suppressed by inserting h-BN between BP and the graphene electrode as a barrier layer, as shown in the right panel of Figure 6c. The insulating h-BN acts as a high-energy barrier [36], while low-energy carriers freed from traps require more energy to cross over this barrier and produce generation–recombination noise. Consequently, h-BN prevents the counter graphene electrode from collecting thermally excited carriers in BP. This can be easily seen from the reduced current amplitude fluctuations. We also extracted the specific detectivity from our devices. We found that the room-temperature detectivity could reach 1.02 × 10^9^ Jones at a 2 V bias, 4 μm wavelength, and 300 K in our heterojunction photo. This detectivity is more than a factor of 100 larger than that of the BP phototransistor control sample.

## 4. Conclusions

In summary, we have presented a few-layer BP/h-BN/graphene heterojunction-based tunneling junction photodetector. The device provides a high photodetection performance at mid-IR wavelengths by facilitating dark noises and the sensitivity of the device is a factor of ~100 larger than that of a BP photo-transistor. Such a great improvement can be attributed to tunneling-assisted noise management. Our novel heterojunction photodetector provides an attractive platform for practical photodetection at long wavelengths, as well as a new strategy for controlling flicker noise.

## Supplementary Materials

The following are available online at https://www.mdpi.com/1996-1944/12/16/2532/s1: Figure S1: Electronic performance of a black phosphorus (BP) device; Figure S2: Noise spectral characterization of a black phosphorus (BP) device; Figure S3: Photocurrent mapping and responsivity of a black phosphorus (BP) device.
