# Supplementary material for: Low-Noise Mid-Infrared Photodetection in BP/h-BN/Graphene van der Waals Heterojunctions
Source: Materials (Basel). 2019 Aug 9;12(16):2532. doi: 10.3390/ma12162532 (PMC6720574; doi:10.3390/ma12162532)
Supplement: Supplementary file 1 [file materials-12-02532-s001.pdf]

materials

MDPI

Supplementary Materials

Low-Noise Mid-Infrared Photodetection in BP/h-BN/Graphene van der Waals Heterojunctions

Qin Lu [1,2,†], Li Yu [2,†] Yan Liu [1,*], Jincheng Zhang [1,*], Genquan Han [1] and Yue Hao [1]

[1] Key Laboratory for Wide Band Gap Semiconductor Materials and Devices of Education, School of Microelectronics, Xidian University, Xi'an 710071, China

[2] School of Electronic Science and Engineering, Nanjing University, 163 Xianlin Ave, Nanjing 210023, China

* Correspondence: xdliuyan@xidian.edu.cn (Y.L.); jchzhang@xidian.edu.cn (J.Z.)

† These authors contributed equally to this work.

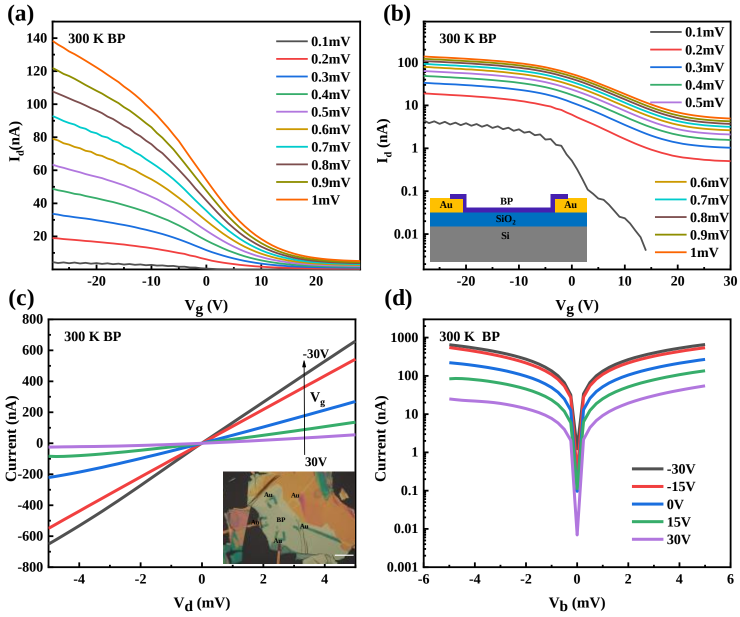

Figure S1. (**a**) Transfer curves obtained from a black phosphorus (BP) device at 300 K with different source-drain voltages (from 0.1 mV to 1 mV). (**b**) Logarithmic current-bias plot of the data in (**a**). The inset is the device schematic on a BP photo-transistor. (**c**) Linear current with two-terminal configuration characteristics at room temperature, demonstrating that the contacts between the metal electrodes and BP flakes are Ohmic. The inset shows an optical image of the BP device. (**d**) Logarithmic current-bias plot of the data in (**c**).

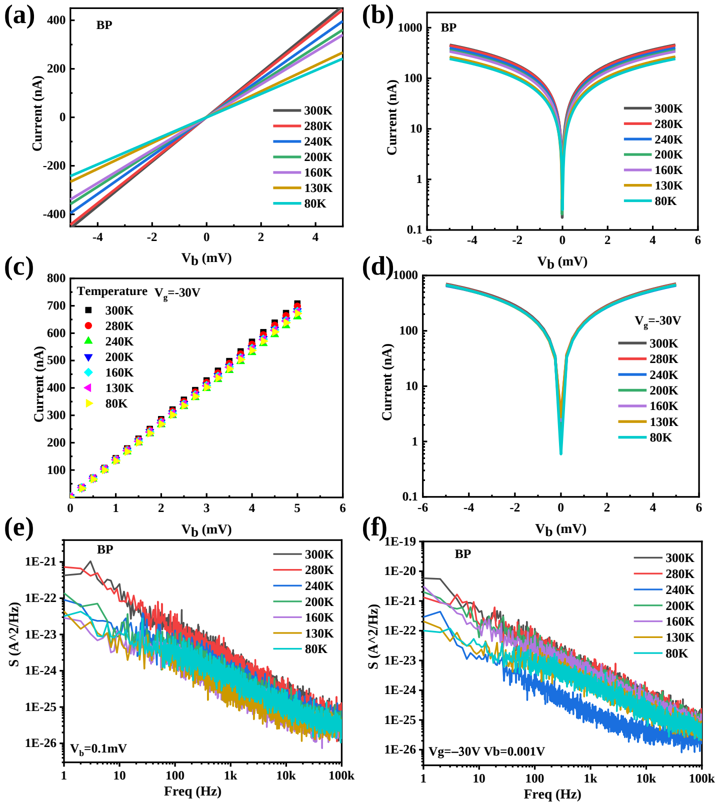

Figure S2. (**a**) Source-drain current as a function of temperature in a back phosphorus (BP) device at zero gate bias. (**b**) Logarithmic current-bias plot of the data in (**a**). (**c**) Source-drain current as a function of the temperature in a BP device at $V_g = -30$ V. (d) Logarithmic current-bias plot of the data in (**c**). (**e**) Noise spectral density $S(I^2)$ in the BP device at various temperatures and 0.1 mV source-drain bias. (**f**) Noise spectral density $S(I^2)$ in the BP device at various temperatures with a −30 V gate voltage and 1 mV source-drain bias.

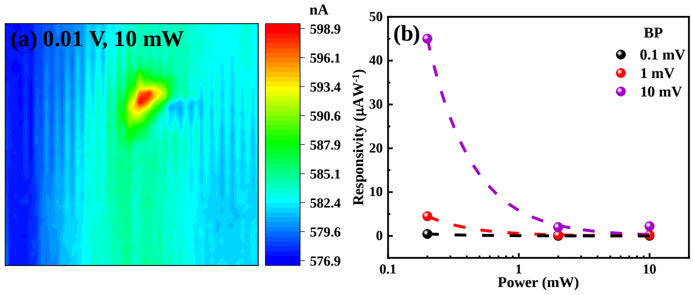

Figure S3. Photocurrent and photoresponsivity measurement in the black phosphorus (BP) device. (**a**) Photocurrent mapping in a BP device at a 10 mW average power and $V_b = 0.01$ V. (**b**) Responsivity as a function of excitation power at various V_b values.
